# The evolution of heat shock protein 90 C-terminal inhibitors: From novobiocin to potential clinical candidates

**DOI:** 10.1016/j.cstres.2026.100165

**Published:** 2026-03-06

**Authors:** Xiaosheng Jiang, Brian S.J. Blagg

**Affiliations:** 1 Department of Chemistry and Biochemistry, University of Notre Dame, Notre Dame, IN 46556, USA; 2 Warren Family Research Center for Drug Discovery and Development, University of Notre Dame, Notre Dame, IN 46556, USA

**Keywords:** Hsp90 C-terminal domain (CTD), Novobiocin, Neuroprotective, Anti-cancer

## Abstract

Heat shock protein 90 (Hsp90) is a highly conserved molecular chaperone that regulates the maturation of various client proteins. Most therapeutic studies have focused on N-terminal Hsp90 inhibitors, but these are limited by dose-escalating toxicities that are caused by induction of the heat shock response. Leonard Neckers’ discovery of novobiocin as a Hsp90 C-terminal inhibitor revealed an alternative mode to Hsp90 inhibition and established the C-terminal domain (CTD) as a therapeutic target. This review highlights recent advances in Hsp90 CTD inhibition and summarizes the evolution of novobiocin-based C-terminal inhibitors. Structure-activity relationship studies are discussed, demonstrating how medicinal chemistry optimization has produced CTD modulators with selective anti-proliferative or neuroprotective activities.

## Background

Leonard Neckers was a pioneer in the field of Heat shock proteins (Hsp's), including Hsp90, and profoundly shaped our understanding of Hsp90 as well as Hsp90 inhibitors. In 2000, his laboratory reported that the natural product and Food and Drug Administration-approved antibiotic, novobiocin, binds a previously unrecognized site in the C-terminal domain (CTD) of Hsp90,^1^, ^2^ thereby revealing an alternative inhibitory mechanism that was distinct from N-terminal Adenosine triphosphate (ATP)-competitive inhibitors—that he also had previously identified. This seminal finding not only challenged the notion that inhibition was confined to the N-terminal Bergerat fold,^3^, ^4^ but also established a foundation for the development of C-terminal inhibitors. By establishing the CTD as a druggable site that avoids induction of the heat shock response, it provided a complementary mechanism to N-terminal Hsp90 inhibition. Neckers’ work catalyzed more than two decades of medicinal chemistry research and mechanistic investigations into the Hsp90 protein folding mechanism, which led to clinical investigation of >20 small molecules. His scientific vision and contributions to the field continues to expand today and serves as an inspiration for the development of new and alternative mechanisms of Hsp90 inhibition, including the novobiocin-derived CTD inhibition discussed in this review.

In the cell, proteins (such as receptors, kinases, enzymes, and more) are the means through which biological processes are orchestrated. Since protein function is determined by its structure, proteostasis is essential for cell viability, as proteins must be properly folded, stabilized, and regulated to maintain survival (proteostasis). Disruptions in proteostasis by external or internal stresses such as acidosis, exposure to toxins, viral infection, DNA damage, metabolic or oxidative imbalances, hypoxia, and heat shock can result in misfolded/denatured proteins, dysregulated cellular activity, and ultimately cell death. To counteract such insults, cells activate protective, pro-survival mechanisms that include the heat shock response, which involves the overexpression of molecular chaperones such as the HSPs to restore protein homeostasis. These molecular chaperones are categorized by molecular weight (e.g., Hsp27, Hsp40, Hsp60, Hsp70, Hsp90, and other large members), wherein each plays a specialized role during proteostasis,^5^^,^^6^

Hsp90 is among the most abundant and highly conserved proteins within the cell and comprises ∼ 2%-3% of total cellular protein in normal cells. However, levels increase to 3%-7% in cancer cells and/or in response to internal/external insults.^7^ This elevated expression correlates with the increased demand for proteostasis in cancer (i.e., an “oncogenic addiction”), whereby rapid proliferation and oncogenic transformation introduce cellular stress and high protein turnover. In fact, Hsp90 is the “master regulator” of the heat shock response and controls induction of the heat shock response.

## Structure and biological functions of Hsp90

**Fig. 1:**
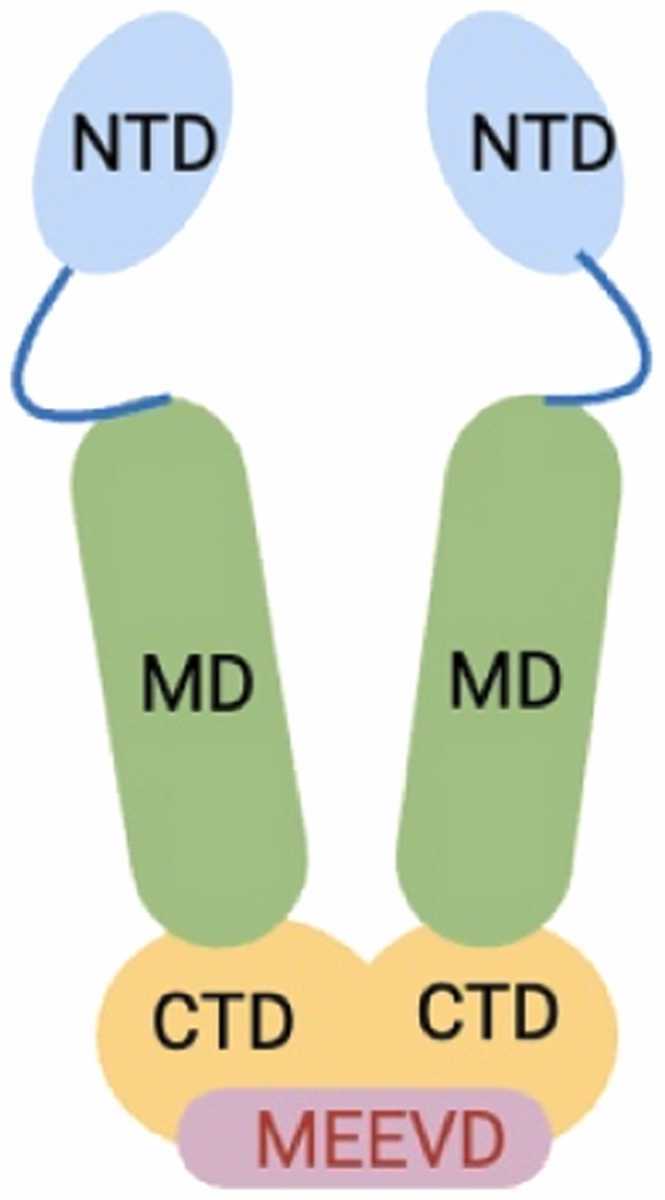
Structure of the Hsp90 homodimer.

Hsp90 is the most abundant Hsp and exists as four distinct cellular isoforms: Hsp90α (inducible) and Hsp90β (constitutively expressed) reside in the cytosol, whereas glucose-regulated protein (Grp94) is located the endoplasmic reticulum, and Hsp75/tumor necrosis factor receptor-associated protein 1 (Trap-1) sequesters to the mitochondria. All four isoforms exist as a homodimer composed of three distinct domains; an N-terminal domain (NTD), a middle domain (MD), and a CTD (Figure 1).^4^, ^8^ The NTD contains an ATP-binding pocket, which contains a structural motif known as a Bergerat Fold that allows ATP to bind in a unique, “C-shaped” conformation. This distinct feature enables selective inhibition of Hsp90 and results in the proteasomal degradation of client proteins, despite the abundance of other proteins that bind/hydrolyze ATP. The middle domain mediates interactions with client proteins and co-chaperones and also regulates ATPase activity. The CTD is responsible for Hsp90 homodimerization,^9^ and contains a nucleotide-binding site that allosterically modulates ATPase activity within the NTD but does not hydrolyze ATP.^10^, ^11^ In addition, the CTD possesses a conserved Met-Glu-Glu-Val-Asp (MEEVD) terminal sequence that is critical for binding co-chaperones that possess a tetratricopeptide-containing repeat domain (TPR).^12^ Interactions with co-chaperones are essential for the regulation and progression of the Hsp90-mediated protein folding cycle.

**Fig. 2:**
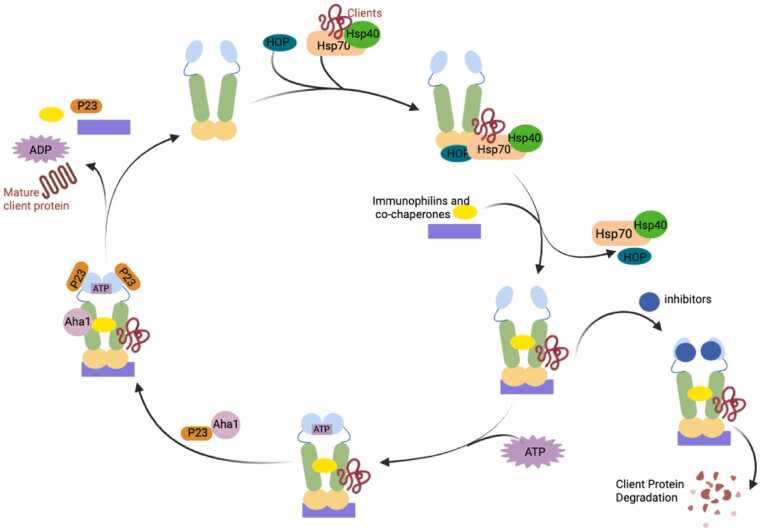
Hsp90 chaperone cycle. Hsp90 transitions through distinct conformational states during its ATPase cycle, which facilitates the maturation and stabilization of client proteins. The cycle begins with the transfer of nascent or partially folded proteins from the Hsp70-Hsp40 complex to Hsp90, mediated by the cochaperone HOP. Additional cochaperones and immunophilins then associate to form a heteroprotein complex with the client protein. Subsequent ATP binding at the N-terminal domain induces N-terminal dimerization and the formation of a closed chaperone complex, a state that is further regulated by cochaperones such as Aha1 and p23, facilitating client protein folding. ATP hydrolysis then triggers Hsp90 to revert to its open conformation, resulting in the release of the folded or activated client protein, followed by dissociation of ADP and recycling of Hsp90 for subsequent folding cycles. However, when an Hsp90 inhibitor binds to either the N- or C-terminal domain, client protein maturation is disrupted, leading to ubiquitination and degradation via the ubiquitin-proteasome pathway. HOP, Hsp70–Hsp90 organizing protein.

Hsp90 and its various co-chaperones/ancillary proteins operate via a complex and as of yet, not fully understood protein folding cycle (Figure 2). During client protein maturation, Hsp90 undergoes a series of dynamic conformational transitions in combination with other proteins to promote the folding and release of substrates. The Hsp90 chaperone cycle can be summarized by the following sequence: Initially, Hsp70–Hsp90 organizing protein and the Hsp70/Hsp40 complex assist in the loading of a client protein onto the Hsp90 homodimer.^13^ Upon delivery, immunophilins and co-chaperones interact with the Hsp90 dimer to assist in folding the polypeptide substrate. When co-chaperones and immunophilins bind, Hsp70, Hsp40, and Hsp70–Hsp90 organizing protein can dissociate to form the activated Hsp90 heteroprotein complex. However, ATP binding and hydrolysis at the NTD along with the association of p23, and other co-chaperones is necessary to promote client protein maturation. After release of the client protein, the Hsp90 heteroprotein complex disassembles, and the Hsp90 homodimer can repeat the catalytic cycle.^14^ (Figure 2) However, when an Hsp90 inhibitor binds to the NTD or CTD, the cycle is terminated and the client is degraded via the ubiquitin-proteosome pathway.^15^ Thus, Hsp90 inhibition turns the normal protein folding machinery into a catalyst for the degradation of substrates, many of which contribute to cancer progression.

## Discovery of Hsp90 inhibitors

### Limitations of N-terminal Hsp90 inhibition

Hsp90 interacts with a wide and diverse range (>400) of client protein substrates to assist in their proper folding, stabilization, and biological activation. These clients are associated with cell signaling pathways that regulate proliferation, metabolism, apoptosis, and transcriptional activity, many of which contribute to the ten hallmarks of cancer.^15^, ^16^, ^17^, ^18^, ^19^ Not surprisingly, Hsp90 inhibitors have attracted intense research since the early 2000s, as inhibition of this molecular chaperone can result in the simultaneous derailment of multiple oncogenic pathways and therefore, manifests an approach similar to combination therapy but via a single biochemical target. Furthermore, Hsp90 belongs to the GHKL (gyrase, Hsp90, histidine kinase, MutL) ATPase superfamily, which is characterized by a distinctive structural motif known as the Bergerat fold. This fold forces ATP to bind in a bent, C-shaped conformation.^3^ Furthermore, the Hsp90 heteroprotein complex present in tumors exhibits a >200-fold increased affinity for ATP^20^ than the homodimer present in unstressed tissue, resulting in a large therapeutic window. The first N-terminal ATP-competitive inhibitors were derived from geldanamycin and radicicol,^21^ two natural products. Neckers identified as the first N-terminal inhibitors. These natural products inspired multiple generations of synthetic analogues, such as 17-N-allylamino-17-demethoxygeldanamycin (17-AAG)^22^, ^23^, ^24^ that induced the degradation of client protein substrates and exhibited antiproliferative activity in vitro. To date, a total of twenty-three Hsp90 N-terminal inhibitors have entered clinical trials.^25^ However, the clinical development of these N-terminal inhibitors has been limited due to unanticipated complications.^25^, ^26^ For example, induction of the pro-survival heat shock response (HSR) resulted in systemic, dose-limiting toxicities during clinical trials for most scaffolds. Since inhibition of the N-terminal ATP-binding site leads to the upregulation of Hsp's, especially Hsp90, drug efficacy is reduced and requires a dose escalation that can result in ocular and cardiotoxic side effects.^25^, ^27^, ^28^ Ultimately, these complications led to the clinical failure of most pan-Hsp90 inhibitors and prompted the development of alternative methods of Hsp90 inhibition to circumvent the detriments associates with N-terminal inhibition.

### Discovery of novobiocin as the first C-terminal inhibitor

**Fig. 3:**
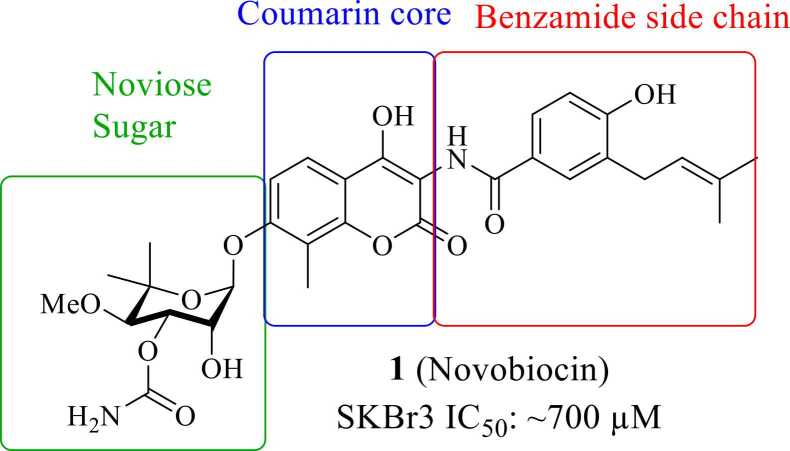
Structure analysis of novobiocin.

In 2000, Leonard Neckers and coworkers made a groundbreaking discovery that changed our understanding of Hsp90 inhibition. Two structurally unrelated natural products, geldanamycin (GA) and radicicol (RD), had previously been shown to bind specifically to an atypical nucleotide-binding pocket located in the N-terminus of Hsp90—a site sharing significant homology with the ATP-binding domain of bacterial DNA gyrase B.^29^ Interaction of these molecules within the NTD disrupted the chaperone function of Hsp90 and destabilized its client proteins. Because the nucleotide-binding site of gyrase B is the target of coumarin antibiotics such as novobiocin (**1**, Figure 3) and the Hsp90 NTD is highly homologous, as both proteins are part of GHKL superfamily^3^, ^29^ (i.e., they both contain the Bergerat fold), the Neckers group hypothesized that novobiocin might also interact with Hsp90. They demonstrated that novobiocin treatment led to a dose-dependent degradation of several Hsp90-dependent client proteins within 16 h in SKBr3 cells, including Raf-1, mutated v-Src, Raf-1, and p185ErbB2 at ∼700 μM.^1^

The authors performed deletion and point-mutation analyzes to identify the binding site responsible for this activity. Intriguingly, mutations within the NTD that abolished GA and RD binding either did not affect or slightly enhanced novobiocin binding, indicating that its binding site was spatially distinct.^1^ Furthermore, systematic truncation of the carboxyl-terminal region identified the novobiocin-binding site to reside within residues 538-728 of Hsp90.^1^, ^30^ Further refinement revealed that deletion of amino acids 657-677 greatly reduced novobiocin binding, while a synthetic peptide corresponding to residues 663-676 competed with full-length Hsp90 for binding to immobilized novobiocin.^1^ Thus, these data indicated that novobiocin binds the C-terminus of Hsp90, which is known to be crucial for both dimerization and interactions with co-chaperones.^30^, ^31^ This finding provided the first evidence that novobiocin could modulate Hsp90 activity through a mechanism distinct from N-terminal inhibition.

Given the dose-limitations associated with Hsp90 NTD inhibition, the CTD has emerged as a mechanistically and structurally distinct target. As previously mentioned, the coumarin antibiotic, novobiocin, was the first compound reported to interact with the Hsp90 CTD by Neckers and coworkers in the early 2000s.^1^ However, subsequent studies revealed that novobiocin inhibits Hsp90 function through an allosteric mechanism,^30^ which explains how this molecule avoids the canonical induction of the heat shock response (HSR) associated with Hsp90 N-terminal inhibitors.

### Distinct regulatory properties of the Hsp90 C-terminal domain

The Hsp90 CTD is known to display chaperone activity that is independent of the N-terminus as well as the dimerization of Hsp90 monomers.^32^ Formation of the C-terminal dimer is essential for Hsp90 function and initiates a complex conformational cycle that enables the reorganization and folding of client proteins. Yun et al suggested that a conformational switch upon novobiocin binding causes changes to Hsp90/cochaperone/client interactions that stimulates dissociation of Hsp90 from the client substrate.^30^ It appears that conformational changes within the C-terminus occur upon inhibitor binding, which result in global structural rearrangements that disrupt the chaperone cycle.

In contrast to N-terminal ATP-competitive inhibition, C-terminal inhibitors modulate Hsp90 function through allosteric mechanisms. These inhibitors bind after dimerization and prevent occupation of the N-terminal ATP-binding pocket. Furthermore, studies by Garnier and co-workers have identified a putative second nucleotide-binding site within the CTD that partially overlaps with the dimerization interface and may adopt a Rossmann-like fold.^1^, ^33^ They concluded that the C-terminal ATP-binding site overlaps with the dimerization domain, thereby explaining the coupling of ATP binding, dimerization, and magnesium-dependent oligomerization. Collectively, these findings highlight the CTD as a mechanistically distinct regulatory domain that governs Hsp90 activity through dimerization and allosteric modulation of the N-terminal ATP-binding site.^34^, ^35^, ^36^ These unique properties distinguish C-terminal inhibition from classical N-terminal ATP-competitive strategies, and therefore, Hsp90 C-terminal inhibition has emerged as an alternative approach to overcome the limitations of N-terminal inhibition.

### Structure-activity relationship insights/studies of novobiocin

**Fig. 4:**
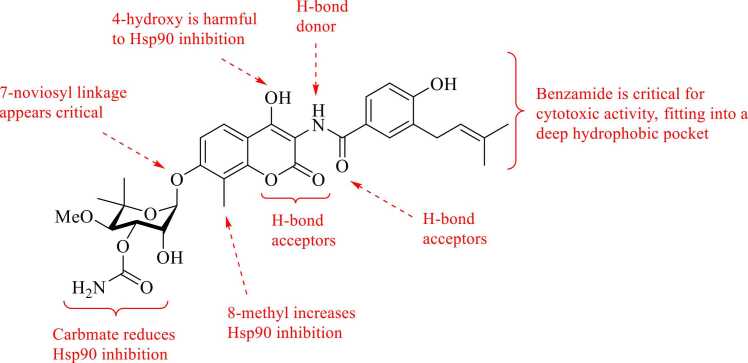
Summary of SAR between novobiocin and Hsp90.

Novobiocin and its derivatives provided the first evidence that selective modulation of the CTD can effectively disrupt Hsp90 function. The novobiocin-based C-terminal inhibitors that have been developed/reported over the years not only serve as valuable chemical probes for elucidating Hsp90 biology, but also serve as promising scaffolds for the development of therapeutics for the treatment of cancer and neurodegenerative diseases. However, the absence of co-crystal structures of small molecules bound to the CTD has severely hindered optimization of these compounds. Consequently, extensive structure–activity relationship (SAR) studies have been conducted to guide the design of potent CTD modulators to overcome the inability to perform rational, structure-based drug design^10^(Figure 4). Structurally, novobiocin contains three modular regions—the benzamide side chain, the coumarin core, and the noviose sugar,^37^, ^38^ each of which has been modified independently to investigate/elucidate Hsp90 SAR.

### Hsp90 in cancer and neurodegenerative diseases

As mentioned earlier in this review, over 400 proteins depend upon the Hsp90 heteroprotein complex to achieve conformational maturation, including tyrosine kinase receptors (eg., EGFR, Her2, and VEGFR), signal transduction proteins (eg., Bcr-Abl, Alk, Braf, and Akt), transcription factors (eg., androgen receptors and HIF1α), cell cycle regulatory proteins (eg., Cdk4, Rb, and cyclin D), and antiapoptotic proteins (eg., Bcl2 and survivin),^39^ many of which are associated with the ten hallmarks. In fact, additional data support that tumor-derived Hsp90 can be targeted selectively over Hsp90 in benign tissues. In cancer cells, Hsp90 exists primarily as a heteroprotein complex that is not only more active than the homodimer found in normal tissue,^15^, ^40^, ^41^ but it also exhibits >200-fold higher affinity for ATP/inhibitors. This observation is further supported by the preferential accumulation of Hsp90 inhibitors in tumors over normal tissue.^42^, ^43^, ^44^, ^45^ Therefore, inhibition of Hsp90 presents a unique opportunity to selectively target cancer cells and simultaneously disrupt multiple oncogenic pathways, a therapeutic strategy similar to combination therapy.^46^

In contrast, Hsp's are beneficial for Alzheimer’s and other neurological diseases/disorders wherein a failure in proteostasis leads to pathological disease. For example, Hsp70 plays a pivotal protective role in multiple disease contexts. Increased expression of Hsp70, a hallmark of the HSR, has been shown to attenuate amyloid-β (Aβ)-induced cytotoxicity and reduce the accumulation of hyperphosphorylated tau proteins.^47^, ^48^, ^49^ Consequently, activation of the HSR is contradictory to anticancer therapy since higher drug concentrations lead to elevated Hsp90 levels, which induces dose-escalating toxicities. However, the upregulation of Hsp70 has been shown to reverse diabetic peripheral neuropathy (DPN) and improve mitochondrial bioenergetics.^50^, ^51^, ^52^, ^53^ Thus, while induction of the HSR exhibits detrimental activities for anticancer activity, it represents a promising cytoprotective mechanism for the treatment of neurodegenerative and metabolic disorders and underscores the versatility of developing small molecule modulators of Hsp90.

## Novobiocin-based scaffolds for Hsp90 C-terminal inhibition

### SAR investigation on the coumarin core

#### Alternate substituents at coumarin C-4 and C-7 position

**Fig. 5:**
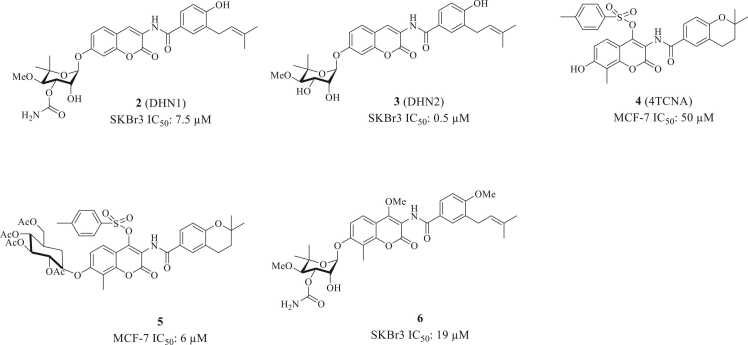
Structures of Initial Novobiocin Analogs with Modifications at C-4 and/or C-7 of the Coumarin Ring.

Initial SAR studies sought to determine which novobiocin moieties are essential for Hsp90 CTD inhibition versus those required for DNA gyrase inhibition. In a seminal study from 2006, the Blagg lab prepared 4-deshydroxy novobiocin DHN1(**2**, Figure 5) and 3′-descarbamoyl-4 deshydroxynovobiocin DHN2 (**3**) and evaluated them for activity against Hsp90. Previous studies demonstrated novobiocin to manifest weak activity against Hsp90, as demonstrated by its ability to induce degradation of ErbB2 in SkBr3 breast cancer cells at ∼700 μM concentration. Therefore, they evaluated both DHN1 and DHN2 by the same procedure. These two compounds showed a marked increase in Hsp90 CTD inhibitory potency as compared to novobiocin. To determine whether the modifications on the coumarin core that resulted in increased Hsp90 inhibition could also produce compounds devoid of DNA gyrase inhibitory activity, they evaluated both DHN1 and DHN2 in a DNA gyrase assay. These results demonstrated that the 4-hydroxyl moiety plays a critical role in DNA gyrase inhibition and that the loss of this group significantly reduces DNA gyrase inhibition ∼200 fold. Whereby, Western blot analyzes showed DHN1 to induce the degradation of both ErbB2 and p53 between 5 and 10 μM, whereas DHN2 induced the degradation of these clients between 0.1 and 1.0 μM, providing evidence that DHN2 is more active than DHN1. Furthermore, these studies established the 4-hydroxyl and 3'-carbamate as critical for DNA gyrase inhibitory activity but detrimental to Hsp90 inhibition.^54^ The importance of the coumarin C-4 position was further highlighted by a 2007 study from the Jack-Michel group. They found that tosylation of the coumarin 4-OH while also removing the noviose sugar yielded an analog dubbed 4-TCNA (4′-tosyl coumarin analog, **4**). 4-TCNA manifested improved antiproliferative activity when compared to novobiocin.^55^ In 2014, the authors introduced an alternative sugar at the 7-position of the coumarin: a protected glucose moiety (7-O-acetyl-glucoside) in combination with the 4-tosyl on coumarin lead compound **5**. Dual substitution at both C-4 and C-7 led to stronger growth inhibitory activity against the MCF-7 breast cancer cell line as compared to 4-TCNA.^56^ Renoir’s SAR studies suggested that modifications at the C-4 and/or C-7 positions, individually or combined, dramatically influence activity, and that the noviose sugar is not necessary if a bulky group like the tosyl substituent is installed at the C-4 position. In 2013, the Gunatilaka group also identified an analog (compound **6**) in which methylation of the 4-hydroxyl group in the coumarin moiety moderately increased biological activity as compared to Novobiocin (Figure 5).^57^

#### Replacing the coumarin scaffold

**Fig. 6:**
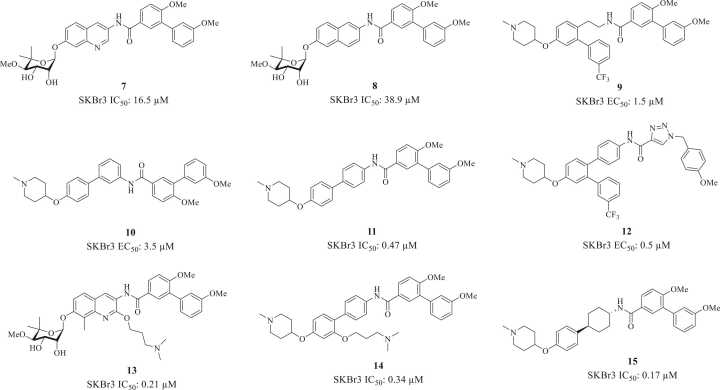
Structures of novobiocin analogs that feature coumarin core surrogates.

Although the coumarin core of novobiocin is a privileged scaffold—“Privileged scaffold” refers to ring systems that are abundant in natural products or commonly found in drugs because they manifest biological activities and/or because they are amenable to structural diversity^58^, ^59^, ^60^—it is not amenable to late-stage diversification. Furthermore, given that the coumarin primarily serves as a linker between the amide side chain and sugar moiety, researchers replaced it with alternative structures to improve drug-like properties. For example, in 2008, Donnelly and coworkers synthesized and evaluated a series of modified coumarin derivatives to further define the essential features of the coumarin core. Analogues **7** (Figure 6), and **8** were the first non-coumarin-based derivatives that contained a quinoline or naphthalene ring in lieu of the 8-methylcoumarin present in novobiocin. Against SKBr3 breast cancer cell lines, both **7** and **8** exhibited greater antiproliferative activities than novobiocin. These results suggested that, while the lactone moiety may provide beneficial hydrogen bonding interactions with the binding pocket, these interactions may not be optimal for antiproliferative activity and support continued exploration of this region of the molecule.^61^ In 2014, Zhao et al prepared compound **9**, which replaced the coumarin ring with a biaryl moiety.^62^ Subsequent studies guided by molecular modeling produced a flexible biphenyl linker (compounds **10** and **11**) in lieu of the rigid coumarin ring system. Remarkably, these biphenyl-based “novologues” inhibited the proliferation of breast cancer cells at midnanomolar concentrations, reflecting an improvement in inhibitory activity. The biphenyl ring’s rotational flexibility appears to allow for an optimal conformation upon binding Hsp90 and perhaps more favorable interactions with the CTD.^63^, ^64^ Moreover, replacement of the coumarin with the biphenyl system simplifies the preparation of additional analogs, wherein each ring system can be evaluated for the rapid elucidation of SARs. In an attempt to further improve potency and to expand the chemical space associated with Hsp90 C-terminal inhibition, it was envisioned that these two molecules could be combined into a single compound, which would exhibit improved interactions than both **9** and **10/11**. Based on this hypothesis, Davis and coworkers introduced the tribenzyl ring system as an alternative scaffold (**12**), which exhibits good inhibitory activity.^65^ Previous SAR studies suggested the potential for an unexplored hydrophilic region about the central core, since the incorporation of an alkylamino side chain onto the central core improved anti-proliferative activity (**13**).^66^ Therefore, it was proposed that placement of this moiety onto the biphenyl core could result in similar improvements. Zhao and coworkers synthesized and evaluated a series of alkylamino biphenylamides and found that incorporation of an alkylamino side chain onto the biphenyl core produced compounds that manifest sub-micromolar to mid-nanomolar inhibitory activity (e.g., **14**). SAR studies revealed that amines attached to a three-carbon linker gave the most potent analogues.^67^ Building upon prior efforts, the biphenyl core was further explored and led to the development of phenyl-cyclohexyl carboxamides as a novel scaffold that exhibited Hsp90 C-terminal inhibition. In fact, some analogs (e.g., **15**) exhibited mid-nanomolar antiproliferative activity across multiple breast cancer cell lines (SKBr3 IC_50_: 0.17 µM; MCF-7 IC_50_: 0.22 µM). The identification of the phenyl-cyclohexyl core represents the most efficacious scaffold discovered thus far for the development of potent and efficacious Hsp90 C-terminal inhibitors.^68^

### SAR studies on the noviose sugar

**Fig. 7:**
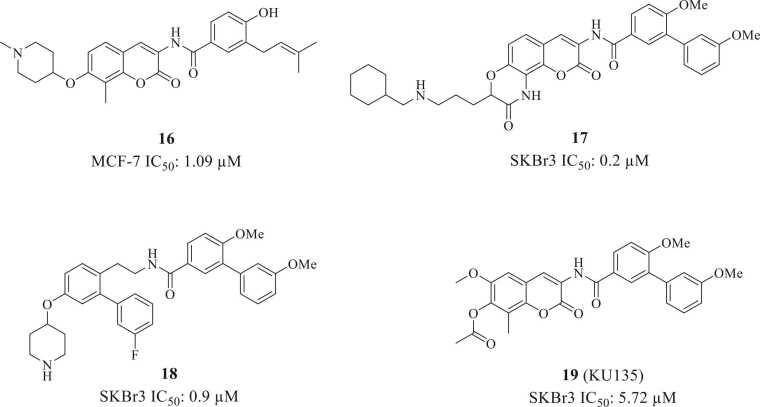
Structures of novobiocin analogs with noviomimetic surrogates.

Given the synthetic challenges associated with preparation of the noviose sugar, researchers initiated SAR studies to identify surrogates that could retain antiproliferative activity while reducing the number of synthetic steps.^69^, ^70^ In 2011, Zhao and coworkers substituted the noviose with various cyclic amines. They determined that replacement of the noviose sugar with a 1′-methylpiperazine (**16**) enhanced anti-proliferative activity^71^ (Figure 7). In 2014, Garg and Zhao designed **17**, an analogue that contains a ring-constrained lactam in lieu of the sugar moiety as a consequence of ring constraint, the analogue was proposed to exhibit a smaller entropic penalty upon binding as compared to their freely rotating counterparts, and could therefore project the side chain into the region normally occupied by the novoise sugar. Furthermore, replacement of the sensitive ether linkage with a lactam would produce analogues with greater stability. The molecules were found to manifest submicromolar to mid-nanomolar anti-proliferative activity.^72^ In 2024, Amatya et al synthesized and evaluated a series of cyclohexyl noviomimetics with varying alkyl chain lengths and electronic properties to probe the size and nature of the Hsp90 CTD binding pocket. From this series, derivatives that contained a piperidine or N-substituted piperidine displayed improved anti-proliferative activities. In addition, the unsubstituted piperidine novologue (**18**) displayed ∼3-fold improved anti-proliferative activity when compared to a boron-based novologue, suggesting the potential for a salt bridge between the positively charged amine (piperidine) and a negatively charged amino acid residue.^73^ In another series, KU-135 (**19**), which contains an acetyl group in lieu of noviose triggered the pro-survival HSR. Consequently, it can serve as an Hsp90 CTD stimulator and induce Hsp70 expression in cells^74^ (Figure 7).

### Benzamide side chain modifications

**Fig. 8:**
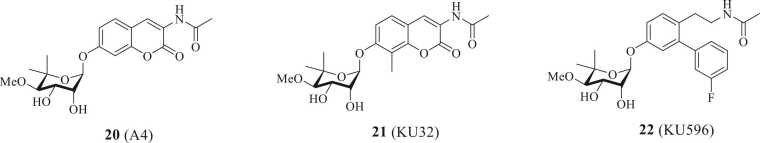
Structures of novobiocin analogs with benzamide side chain modifications that display neuroprotective activity.

The amide side chain is another critical determinant of potency for Hsp90 C-terminal inhibitors. The first improved novobiocin analogue reported was A4 (**20**) (Figure 8), which shortened the amide side chain to an acetamide group and induced the degradation of Hsp90 client proteins such as AKT, Her2, and Hif-1α at a ∼ 1 μM dose.^69^ However, in contrast to the analogs containing an aryl amide side chain, A4 induced Hsp90 expression at concentration < 0.1 μM in LNCaP cells.^75^ Therefore, an SAR study on the amide side chain was pursued and demonstrated that a short alkyl or cycloalkyl amide side chain induced Hsp levels at significantly lower concentrations than that needed to induce client protein degradation, which suggested these compounds may be useful for neuroprotection.^76^ It was determined that more than five carbons on the amide side chain diverts the modulator from a neuroprotective agent to an antiproliferative agent.^77^ Subsequent studies led to KU32 (**21**) and eventually KU596 (**22**) (Figure 8) as neuroprotective agents.^53^, ^76^ Studies have demonstrated that they exhibit potent neuroprotective activity by induction of pro-survival HSR at a sub nanomolar concentrations.^53^, ^78^

**Fig. 9:**
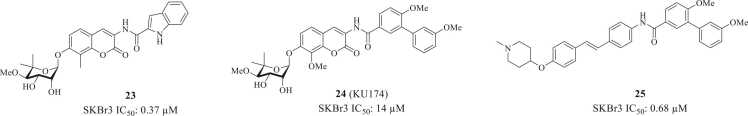
Structures of novobiocin analogues with benzamide side chain modifications that display anticancer activity.

Based on these results, the Blagg group focused on modification of the side chain to improve cell-based anticancer activity via a series of analogues containing various aryl amides. There were two notable studies between 2008 and 2011. One of them produced compound **23** (Figure 9), which contains a 2-indoleamide and led to an increase in antiproliferative activity against multiple cancer cell lines (SKBr3 IC_50_: 0.37 µM; MCF-7 IC_50_: 0.57 µM; HCT-116 IC_50_: 0.17 µM etc.).^79^ Another notable compound is KU174 (**24**), which presents a biaryl amide side chain. KU174 exhibits anti-proliferative and cytotoxic activity along with client protein degradation and disruption of Hsp90 native complexes without induction of the HSR.^80^ Subsequent studies in 2017 determined the optimal distance and angle between the N-methylpiperidine and biaryl amide side chain. Compound **25** exhibited good anti-proliferative activity against multiple cancer cell lines that paralleled Hsp90 inhibitory activity. These studies revealed that the sugar and amide moieties should be between 7.7 and 12.1 Å apart along at an angle of 180°.^81^ In an effort to elucidate SARs for the amide, Amatya and coworkers designed and synthesized a series of Hsp90 C-terminal inhibitors containing various amide bioisosteres. The data indicated that a secondary amide is necessary for Hsp90 binding/activity, as it presents a hydrogen bond and/or maintains proper geometry within the binding site. Replacement or removal of the amide linkage decreased antiproliferative activity in most examples, and none of the bioisosteres proved more efficacious than the lead compound.^82^

## Clinical status

One of the most advanced novobiocin analogues is KU-596, also known as RTA 901 (cemdomespib), and is currently undergoing clinical trials. As a neuroprotective agent, RTA 901 leveraged the unique HSR-inducing, pro-folding activity of KU-596 to treat diabetic peripheral neuropathy. It became the first Hsp90C-terminal inhibitor to enter clinical trials for the treatment of diabetic neuropathy by Reata Pharmaceuticals. This compound manifested excellent safety in preclinical models, protecting neurons from misfolded protein stress without toxicity. Reata Pharmaceuticals advanced RTA 901 through Phase 1 clinical trials, wherein it demonstrated a good safety and pharmacokinetic profile in healthy volunteers. At the end of 2021, RTA 901 completed Phase 1 and entered Phase 2 trials for diabetic neuropathy (**ClinicalTrials.gov ID** NCT05895552). However, no CTD inhibitors have yet progressed to clinical trials for the treatment of cancer. Despite potent in vitro antiproliferative activities, challenges remain. A major hurdle that has yet to be overcome is the lack of a co-crystal structure of Hsp90’s CTD bound to an inhibitor. This absence of high-resolution structural insight makes structure-based design difficult. Nevertheless, research is ongoing to optimize these compounds, and it is expected that new compounds will be developed with improved biological activities.

## Conclusion

Inhibition of the Hsp90 CTD offers a distinct set of regulatory features and potential therapeutic advantages. C-terminal ligands can differentiate neuroprotective activity from anticancer activity, leading to selective degradation of oncogenic client proteins without induction of the HSR that is associated with N-terminal Hsp90 inhibition. This review summarizes the structure and functional role of the Hsp90 CTD and provides a comprehensive review of novobiocin-based C-terminal modulators, beginning with Neckers’ pioneering discovery that novobiocin binds a regulatory region within the Hsp90 C-terminus. Subsequent medicinal chemistry efforts have transformed novobiocin from a weak natural product Hsp90 inhibitor into optimized CTD modulators. Although the absence of co-crystal structures has posed challenges for the development of more potent inhibitors, substantial progress has been made toward elucidating SARs and advancing CTD modulators as both chemical probes and therapeutic leads.

## Future perspective and potential application

Looking forward, future research on Hsp90 CTD inhibition may benefit from alternative strategies that enhance target engagement while preserving the unique regulatory advantages of CTD modulation. One potential direction is the development of covalent CTD inhibitors that exploit reactive cysteine residues within the CTD. Li et al identified a cysteine residue (Cys598) within the Hsp90 CTD that is positioned adjacent to an exposed and potentially druggable binding site that can be targeted for covalent modification.^83^ In parallel, continued efforts to obtain high-resolution co-crystal structures of the Hsp90 CTD bound to ligands would greatly facilitate a structure-guided approach. Structural insights into ligand binding modes, allosteric conformational changes, and the spatial relationship between druggable pockets and reactive cysteine residues would enable a rational optimization of molecular potency and selectivity, thereby accelerating the development of next-generation CTD inhibitors.

Hsp90 CTD inhibitors exhibit broad applications across both cancer and non-cancerous indications. In oncology, CTD-targeting strategies are particularly promising for tumors driven by Hsp90-dependent oncoproteins, including breast cancer,^2^, ^84^ prostate cancer,^80^, ^85^ non-small cell lung cancer,^86^ leukemia,^87^ and melanoma,^88^ wherein destabilization of client proteins can be achieved without induction of the HSR. Beyond cancer, CTD modulation offers additional opportunities to treat diseases that are characterized by a proteostatic imbalance, such as neurodegenerative disorders.^53^, ^76^, ^77^ Emerging evidence also supports exploratory applications in inflammatory,^89^ metabolic,^90^ and viral diseases.^91^ Collectively, these findings underscore the versatility of Hsp90 CTD inhibitors as both therapeutic candidates and as chemical tools for selectively modulating chaperone biology across diverse pathologies.

## CRediT authorship contribution statement

**Xiaosheng Jiang:** Writing – original draft, Data curation. **Brian S.J. Blagg:** Writing – review & editing, Supervision, Conceptualization.

## Declarations of interest

Brian Blagg is an inventor of KU-596/Cemdomespib, which is currently undergoing clinical evaluation.

## Data Availability

Data will be made available on request.
